# A New Practical Method of Fusing Platinum

**Published:** 1894-05

**Authors:** L. E. Custer

**Affiliations:** DAYTON, O.


THE
AMERICAN JOURNAL
—OF—
DENTAL SCIENCE.

VOL. XXVIII. BALTIMORE, MAY, 1894. No. 1.

ARTICLE I.

### A NEW PRACTICAL METHOD OF FUSING PLATINUM.

BY L. E. CUSTER, B. S., D. D. S., DAYTON, O.

Read before the Ohio State Dental Society, Dec. 1893.

The process I am about to describe is one in which electricity is the active agent, so far a clear understanding, technical terms will be avoided as much as possible. Although the perfected process is quite simple, it is the result of considerable study and experimentation.

The production of heat by electricity depends upon two factors, the quantity of electricity and the resistance of the conducting agent. As the quantity is increased the heating power is increased, but this power is not apparent until the current meets with some resistance. The unobstructed flow of any quantity of the fluid does not produce heat. It is only when there is placed in the circuit a poor conductor of electricity that we have this manifestation.

All metals are comparatively good conductors of electricity, yet these vary in their conducting power. Copper stands at one extreme and German silver at the other. In the electric gold annealer, platinum wire is used because it is a poor conductor and does not oxidize. (Experiment with copper and German silver wire.)

The size of the wire is another factor in the determination of heat. With a given length of wire the resistance increases as the diameter of the wire decreases. That is, a small wire has less carrying capacity than a large one, so that when the same amount ot current that is easily conducted by the large one, is forced through a small wire, the condensation, I will term it, produces heat. (Experiment with large and small wire.) So with the same quantity of electricity at a given pressure, heat is produced according to the resistance of the conducting agent.

The atmosphere is practically a non-conductor of electricity, yet under certain circumstances it does allow the passage of an electric current. When a current has been established by bringing two terminals together, the electricity continues to flow, even after the ends have been separated. It leaps the intervening thickness of air in the form of the voltaic arc and continues until this distance becomes too great. (Experiment with carbons). This action develops so much heat that it has led to the supposition that electricity is another form of heat. When the oppositely electrical clouds unite, it passes through the atmosphere as lightning, when the leyden jar discharges, it is the spark, or when it lights the street corner, it is the arc. The heat of the arc is so intense that by our present knowledge it cannot be measured. By the ordinary incandescent current I find that we can only melt platinum and iridium but in small quantities we can vaporize them.

Upon the foregoing I have devised a practical method for making and using the voltaic arc for melting metals of a high fusing point. While it is equally applicable to the "arc current" so called, and 500 and 220 volt currents, I will describe the appliance as adopted to the 110 volt current, that which is used for incandescent lighting and which is the ideal current for dental purposes.

Since a large quantity of current is necessary, the safety plugs should be as large as No. 16 or 18, English standard gauge. A resistance coil of four pounds of No. 18 copper wire will prevent fusing the plug and at the same time give a large arc. This is placed at a convenient point in the circuit. It becomes heated, so should be insulated and ventilated on asbestos if it is to be used any length of time.

A block of carbon, such as is used in batteries, is connected with one wire for the receptacle, and a carbon pencil is attached to the other wire. Carbon is used for the receptacle because it is a conductor of electricity, a poor conductor of heat, is non-combustible, and can easily be fashioned to mould the melted metal. An improved bed is composed of ordinary stove blacking and powdered asbestos, equal parts, and thoroughly mixed and united. This is a poor conductor of electricity and is itself heated by the current. It is also a better non-conductor of heat, which is of importance where large quantities are to be melted. A piece of charcoal being a conductor of electricity and a poor conductor of heat, also makes an excellent receptacle. This part of the appliance should be mounted on an asbestos base, or if small quantities are to be fused, it may be held in the left hand by a suitable handle.

In the right hand the carbon pencil is to be used. This is made of an electric light carbon five or six inches long. A hole is drilled two thirds its length, and in this hole is inserted the other terminal wire. This wire is so insulated that only the end comes in contact with the carbon. By this arrangement the upper two-thirds of the pencil, although charged electrically, does not become heated and answers for a handle. With the incandescent current there is no danger to life.

The operation is very simple. The platinum is laid on the carbon bed and the pencil is brought in contact with it. Immediately there is a current established from the pencil through the platinum to the carbon bed or *vice versa* Upon raising the pencil a short distance an arc is formed directly upon the metal and it is melted. The arc can be carried about at will until the pieces are all brought into one mass.

With the above arrangement from five to eight dwts. may be melted at once, but there is no limit to the amount that can be gotten together in one mass by a process of fusing. It is possible to so manipulate the arc that only part of the mass will be melted and to this more metal added.—*Dental Register*.

